# Arthroscopic Management of Medial or Rotational Ankle Instability: A Comprehensive Review of Current Evidence

**DOI:** 10.3390/healthcare13121398

**Published:** 2025-06-11

**Authors:** Chiara Barbieri, Guido Bocchino, Daniele Grassa, Doriana Di Costa, Elena Gabrielli, Fabrizio Forconi, Giulio Maccauro, Raffaele Vitiello

**Affiliations:** 1 Department of Orthopedics and Geriatric Sciences, Catholic University of the Sacred Heart, 00168 Rome, Italy; chiara.barbieri04@icatt.it (C.B.); guido.bocchino01@icatt.it (G.B.); daniele.grassa01@icatt.it (D.G.); doriana.dicosta01@icatt.it (D.D.C.); elena.gabrielli01@icatt.it (E.G.); giulio.maccauro@policlinicogemelli.it (G.M.); raffaele.vitiello@policlinicogemelli.it (R.V.); 2 Department of Orthopedics, Ageing and Rheumatological Sciences, Fondazione Policlinico Universitario A. Gemelli IRCCS, 00168 Rome, Italy

**Keywords:** medial ankle instability, arthroscopy, deltoid ligament, ligament repair, rotational ankle instability, ankle sprain, systematic review

## Abstract

Introduction: Rotational ankle instability (RAI), involving combined medial and lateral ligament insufficiency, is an increasingly recognized clinical entity. While open surgery has traditionally been the mainstay for treating deltoid ligament injuries, recent developments in arthroscopic techniques offer a minimally invasive alternative. This systematic review aimed to evaluate the current evidence on the arthroscopic management of medial and rotational ankle instability, focusing on surgical techniques, clinical outcomes, and complications. Methods: A systematic literature search was conducted following PRISMA guidelines using the PubMed, Scopus, and Web of Science databases. The search strategy included the following terms: ((rotation instability) OR (deltoid) OR (medial ankle instability)) AND (ankle arthrosc*). Eligible studies included adult patients undergoing arthroscopic repair of medial ankle instability with a mean 26.4 months follow-up and reported clinical outcomes. Ten studies met the inclusion criteria, encompassing 336 patients and 346 ankles. Results: The mean patient age was 32.6 ± 5.0 years, with 80.6% being male. MRI was the primary diagnostic tool across most studies. Ankle sprains were the most common cause of instability. Lateral ligament insufficiency was frequently associated with medial injuries, reported in all studies evaluating this parameter. All patients underwent prior conservative treatment (mean duration: 5.6 months). Surgical management involved all-inside arthroscopic repair using knotless suture anchors. Additional procedures were performed in 90% of studies, including osteophyte resection (33.3%) and microfracture (22.2%). The mean follow-up period was 26.4 months. The mean postoperative AOFAS score was 95.3, with return to sport generally achieved between 3 and 5 months. Complications were minimal, primarily consisting of superficial wound issues and transient nerve irritation; no major complications or revision surgeries were reported. Discussion: Arthroscopic management of medial and rotational ankle instability is associated with excellent functional outcomes, low complication rates, and early return to sport. Compared to open procedures, arthroscopic techniques offer advantages including reduced soft tissue trauma, fewer wound complications, and the ability to address concomitant intra-articular lesions in a single session. Although technically demanding, this approach is particularly beneficial in athletic populations. However, high-quality prospective studies are still needed to validate these findings and establish long-term comparative outcomes with open reconstruction techniques.

## 1. Introduction

Ankle sprains are among the most common musculoskeletal injuries, primarily affecting the lateral ligament complex of the ankle joint. These injuries impact all age groups, especially athletes and active individuals [1]. The annual incidence is 2–7 per 1000 individuals, though many cases go unreported [2].

The most frequent cause of injury is an involuntary inward twisting of the ankle. The ligaments most affected are the anterior talofibular ligament (ATFL), followed by the calcaneofibular ligament (CFL). While most acute ankle sprains respond well to non-surgical treatment, around 15–20% of individuals continue to experience symptoms and may go on to develop chronic ankle instability (CAI), leading to recurring sprains [3,4,5].

The medial collateral ligament (MCL) of the ankle or deltoid ligament is the primary stabilizer against valgus forces to the ankle, and it is subject to acute or chronic injuries. The mechanism of injury leading to acute MCL damage involves a force in pronation and external rotation or, less commonly, in supination and external rotation [6]. Isolated MCL injuries without fractures are rare but documented [7,8].

CAI involves mechanical instability, recurrent sprains, and proprioceptive deficits [9,10]. The diagnosis of chronic ankle instability requires an integrated approach. Functional assessments, including the anterior drawer test and inversion stress test, are essential for identifying ligamentous laxity [11,12]. Imaging modalities, such as radiography, ultrasound, and MRI, further enhance diagnostic accuracy [13]. Patients with CAI may have both medial and lateral ankle symptoms [14].

Deltoid ligament alterations have been reported in individuals with chronic ankle instability (CAI) [15,16,17]. Approximately 40% of these patients exhibit partial injury to the deltoid ligament [18]. The notion of rotational ankle instability (RAI) refers to a combined disruption of the anterior talofibular ligament (ATFL) and the deltoid ligament, particularly its anterior fibers, which carries significant therapeutic and prognostic relevance according to Barbosa-Torres [19]. Under weight-bearing conditions, disruption of the ATFL is associated with increased anterior displacement, internal rotation, and upward migration of the talus [20], contributing to the pathogenesis of degenerative joint disease. The deltoid ligament may be affected by trauma or degenerative changes secondary to altered talar mechanics induced by ATFL insufficiency [21].

RAI should be considered in cases involving lesions of the anterior deltoid ligament fibers and the ATFL. Its clinical identification is often challenging, especially in patients without medial symptoms. When only lateral ligament reconstruction is performed, unrecognized medial instability may become evident postoperatively. A thorough assessment of the deltoid ligament is therefore recommended, and if injured, it should be addressed during the same surgical procedure, particularly in the presence of persistent medial-sided symptoms or suspected RAI.

In line with this approach, the growing interest in addressing medial ankle instability has led to the development of arthroscopic techniques targeting MCL ligament injuries [22], with recent studies reporting excellent outcomes, including improvements in American Orthopaedic Foot and Ankle Society (AOFAS) scores and subjective ankle stability [23].

However, few techniques have been specifically described for the arthroscopic treatment of medial ligament insufficiency, and none have focused exclusively on isolated or predominant medial instability.

This review aims to provide an up-to-date overview of the current evidence regarding the arthroscopic management of medial or rotational ankle instability. Particular attention is given to the role of arthroscopy in the diagnosis and treatment of deltoid ligament injuries, as well as to the challenges and future perspectives in this evolving field.

## 2. Materials and Methods

The review followed PRISMA (Preferred Reporting Items for Systematic Reviews and Meta-Analyses) guidelines [24], ensuring a thorough and systematic approach to data collection and analysis. This systematic review has also been registered with the International Prospective Register of Systematic Reviews (PROSPERO), under registration number CRD420251027750.

### 2.1. Search Strategy

The search was performed across several online databases, including PubMed, Scopus, and Web of Science. The search string used was as follows: ((rotation instability) OR (deltoid) OR (medial ankle instability)) AND (ankle arthrosc*).

We carefully examined the titles and abstracts of all retrieved articles to assess their eligibility for inclusion in the review. The criteria for inclusion were as follows: the studies must involve human adults, be published in English, and have publication dates up to January 2025. We included randomized trials, uncontrolled comparative trials, and case series. When there was uncertainty, the full article was retrieved for further examination. The senior author and the content area experts then obtained the full text of all articles and reviewed them to minimize any bias that could arise from preconceived opinions about the studies and their findings. This process was further enhanced by following up on the reference lists of relevant studies to identify additional articles.

Two authors (G.B. and C.B.) independently reviewed the abstracts, obtaining the full texts for any abstracts that were inconclusive. Any differences between the reviewers were discussed, and if disagreements remained, the senior author (R.V. or G.M.) was consulted. The reference lists of the selected articles were manually checked to identify additional relevant studies. All selected studies were then analyzed retrospectively by three authors (D.D.C., G.B. and D.G.), who extracted and entered the data into an Excel worksheet. Finally, the data sheet was reviewed by four authors (R.V., E.G., C.B. and D.G.), who reached consensus on the extracted data. Additionally, the references of the identified papers were searched to find further relevant articles, and all journals were considered.

### 2.2. Inclusion and Exclusion Criteria

The eligibility criteria for our analysis were established to include studies that met high methodological and reporting standards in the evaluation of arthroscopic management of medial ankle instability. We included therapeutic clinical studies that specifically evaluated arthroscopic medial ankle ligament repair, with a minimum follow-up period of 12 months for all patients. Eligible studies were required to report preoperative and postoperative outcome scores, be published in peer-reviewed journals, and be written in English. Furthermore, only studies with a full-text version available were considered.

To maintain the quality and clinical relevance of our analysis, we excluded certain types of studies. These included review articles, case reports, technique articles, cadaveric studies, animal studies, and in vivo basic science research. Additionally, studies that did not provide outcome scores or lacked sufficient follow-up data were excluded (Table 1).

Three reviewers (G.B., C.B. and D.G.) independently conducted the screening and review process, evaluating the full texts of the selected articles to assess their eligibility and extract relevant data. In cases of uncertainty regarding study inclusion, the final decision was made by the senior author. Additionally, three authors (D.D.C., E.G. and C.B.) independently assessed the risk of bias using standardized evaluation criteria. Any disagreements were resolved through discussion, and when needed, a supervising author (R.V.) was consulted to reach consensus.

**Figure 1:**
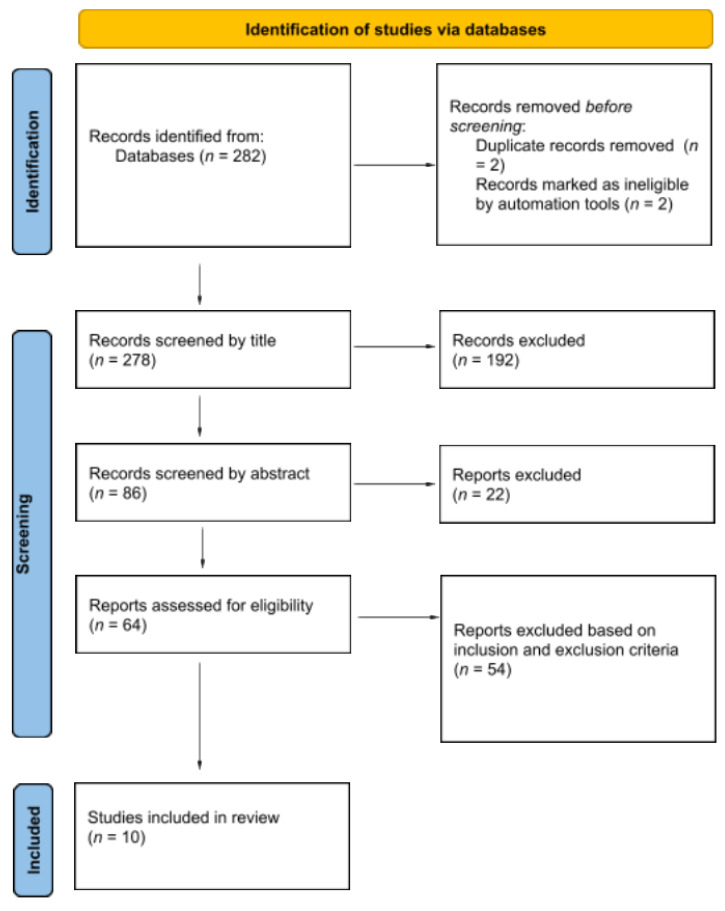
PRISMA flowchart.

A total of 282 articles were initially identified through database searching. After removing 2 duplicates and 2 ineligibles using automation tools, 278 records were screened by title and abstract. Of them, 214 were excluded. The remaining 64 full-text articles were assessed for eligibility. Fifty-four studies were excluded based on the inclusion and exclusion criteria. As a result, 10 studies were included in the final qualitative synthesis. The selection process is detailed in the PRISMA flowchart (Figure 1).

### 2.3. Data Extraction and Analysis

The titles and abstracts were independently screened by two reviewers, G.B. and C.B. For abstracts that either met the inclusion criteria or caused uncertainty, full-text articles were obtained. These full texts were subsequently re-evaluated by the same two independent reviewers. Any discrepancies were resolved through assessment by the senior author, R.V.

The methodological quality of each study was assessed using the Methodological Index for Non-Randomized Studies (MINORS) score, which offers a maximum of 24 points for comparative studies and 16 points for non-comparative studies [12]. Two authors, R.V. and E.G., independently assigned MINORS scores and reached consensus on the final score.

Statistical significance was determined at a threshold of *p* < 0.05. The gathered data were analyzed and organized using SPSS 26 software (SPSS, Inc., Chicago, IL, USA). Categorical variables are displayed as frequencies and percentages, while continuous variables are presented as means with their standard deviations. All numerical data have been rounded to one decimal place for enhanced precision.

### 2.4. Extracted Variables and Outcomes

From each included study, we extracted data on demographic and clinical variables, surgical techniques, outcomes, and complications. Demographic variables included patient age, sex, and athletic status, while clinical variables included the mechanism of injury and whether the injury was unilateral or bilateral. Although laterality (left or right ankle) was considered a relevant variable, it was not consistently reported across studies and was therefore excluded from the analysis.

We also recorded the type and location of ligamentous injury (e.g., anterior talofibular ligament or deltoid ligament), the presence of isolated or combined medial/lateral instability, and the specific surgical technique performed, such as isolated arthroscopic deltoid repair or combined medial–lateral repair.

The primary clinical outcome of interest was postoperative functional recovery, most commonly measured using the American Orthopaedic Foot and Ankle Society (AOFAS) score. Both preoperative and postoperative values were extracted when available, and outcomes were summarized as the mean ± standard deviation or reported ranges. Secondary outcomes included the time to return to sport, recurrence of instability, subjective perception of ankle stability, and the presence and type of postoperative complications. Complications were extracted as reported in each study. Since the original articles did not consistently differentiate between major and minor complications, we reported all adverse events without subclassification.

Where comparative data were available, such as in studies reporting subgroups (e.g., with vs. without deltoid repair), we noted relevant effect estimates, including statistically significant differences in return-to-sport time (*p* = 0.03). However, most included studies did not provide formal estimates of relative effect, such as risk ratios or hazard ratios. Given the heterogeneity in study design and outcome reporting, we performed a qualitative synthesis of the data.

## 3. Results

### 3.1. Patient Demographics

A total of 10 studies were included, comprising 289 patients and 291 ankles treated for medial or rotational ankle instability with arthroscopic techniques. The number of patients per study ranged from 7 to 81, with a median sample size of 25 patients. The mean age across studies was 32.6 ± 5.0 years, with reported age ranges spanning from 23 to 40.1 years. Sex distribution was available in 9 out of 10 studies. Among them, 155 were male (54.8%) and 128 were female (45.2%), reflecting a predominance of male patients in this cohort (Table 2).

In the subset of studies that reported occupation or athletic status, a significant proportion of patients were identified as competitive or recreational athletes, particularly in high-impact sports such as football (*n* = 5) and basketball (*n* = 2). However, the majority of studies did not systematically report activity level or occupational exposure.

### 3.2. Diagnosis and Cause of Instability

The diagnosis of medial ankle instability was established using both clinical and imaging assessments. The most commonly used diagnostic tool was magnetic resonance imaging (MRI), which was employed in the majority of studies to evaluate the integrity of the deltoid ligament and identify associated lesions such as osteochondral defects or syndesmotic injuries. Clinical stress tests, including the medial drawer and valgus stress test, were also described, although in some cases, instability was only confirmed intraoperatively through arthroscopy [33].

Regarding the etiology of instability, ankle sprain was the most frequently reported cause, mentioned in at least five studies. Specifically, eversion sprains were documented in two studies, while supination–external rotation trauma was reported in one study involving 22 patients. One study described multiple severe sprains as the primary mechanism. Additionally, 16 fractures, 1 anterior dislocation, and 4 cases of malleolar fracture complications were identified as contributing factors in a mixed cohort. Other reported causes included chronic ankle instability and residual instability after previous lateral ligament repair, highlighting the heterogeneity and complexity of rotational and medial ankle instability.

### 3.3. Associated Conditions

Lateral ankle instability was frequently reported in conjunction with medial instability. Among the ten studies that specifically addressed this parameter, **six** confirmed the presence of lateral ligament insufficiency, either through clinical examination or intraoperative findings. In some cases, medial instability only became clinically evident after lateral ligament repair, supporting the hypothesis that medial symptoms may be masked by dominant lateral dysfunction. Overall, lateral ligament involvement was consistently (100%) observed across all studies on RAI, underlining the necessity of a comprehensive evaluation of both ligamentous structures in the diagnostic and therapeutic process.

Regarding associated intra-articular and periarticular conditions, seven studies described additional pathologies. The most common findings were os trigonum (reported in four patients), anterior tibial osteophytes (two cases), osteochondral lesions of the talar dome (two cases), loose intra-articular bodies (one case), and isolated calcaneofibular ligament injuries (one case). On average, studies that reported associated conditions documented 1.7 concomitant pathologies per study, underlining the complexity of these cases. These findings further highlight the importance of comprehensive preoperative imaging and arthroscopic exploration to identify and appropriately manage all contributing lesions.

### 3.4. Management

All included studies (100%) reported an initial attempt at conservative management before proceeding to surgical treatment. In total, 10 out of 10 studies explicitly described a non-operative approach as the first-line treatment. Conservative management typically included kinesiotaping [34], physical therapy, proprioceptive training, muscle strengthening [35], and in some cases, the use of orthotic support [36].

The average duration of conservative treatment, when specified, was approximately 5.6 months, with reported durations ranging from a few weeks to over six months. Surgery was generally indicated in cases where patients experienced persistent mechanical instability, pain, or recurrent sprains and failed to return to pre-injury activity levels despite an adequate rehabilitation program (Table 3).

### 3.5. Surgical Treatment and Additional Procedures

All studies included in this review employed arthroscopic surgical techniques for the management of medial or rotational ankle instability. The most adopted approach was an all-inside arthroscopic repair with knotless suture anchors, targeting the anterior fascicles of the deltoid ligament. This technique was frequently combined with lateral ligament repair in cases of rotational or combined instability.

In addition to standard repair, ten cases of retensioning procedures were reported in one study, and augmentation with tendon grafts, including the plantaris tendon, was described when native ligament tissue was insufficient. These strategies were applied selectively, based on intraoperative assessment of ligament integrity.

Importantly, 9 out of 10 studies (90%) reported the execution of at least one additional surgical procedure to address concomitant pathologies identified during arthroscopic exploration. Among them, osteophyte resection was the most frequent, performed in 33.3% of studies with additional interventions. Microfracture procedures for talar osteochondral lesions were also common, occurring in 22.2% of cases. Other reported procedures included peroneal tendoscopy, syndesmosis fixation, lateral internal bracing, and posterior or hindfoot endoscopy, although these were less frequently detailed (Table 3).

These data underscore the frequent coexistence of secondary intra-articular abnormalities in patients with medial or rotational ankle instability and highlight the role of arthroscopy in allowing both precise diagnosis and comprehensive treatment in a single operative setting.

### 3.6. Post-Operative Protocol

Post-operative rehabilitation protocols were reported in all studies, although with some variations. Most patients were initially immobilized in a walker boot and remained non-weight-bearing for 1 to 3 weeks, followed by progressive partial weight-bearing. Physical therapy was typically initiated between the first and second post-operative week, and included range-of-motion, proprioceptive, and strengthening exercises. The average follow-up period across the included studies was 26.4 months, ranging from 12 to 52 months. Return to sports was generally allowed between 3 and 5 months, depending on clinical progress and functional recovery.

### 3.7. Clinical Outcomes

Functional outcomes were assessed in all included studies using the American Orthopaedic Foot and Ankle Society (AOFAS) score. The overall mean postoperative AOFAS score was 95.3, with reported values ranging from 86 to 100. Mansur et al. [25] reported a mean score of 91.9 at 14.8 months of follow-up. Vega et al. [23,26,31] reported a score of 100, with follow-up durations of 35, 34, and 22 months, respectively. Buchhorn et al. [19] reported a score of 94.1 at 12 months, Seiça et al. [27] reported a score of 86.9 at 21.3 months, and Li et al. [28] reported a score of 98.0 at 28 months. Hintermann et al. [30] reported a score of 91.6 at 52 months. Lewis et al. [29] and Guelfi et al. [32] did not report AOFAS scores numerically but described favorable outcomes. In one comparative study, return to sport occurred earlier in patients who underwent deltoid ligament repair compared to those managed without repair (4.6 ± 1.6 vs. 6.0 ± 2.5 months; *p* = 0.03), with similar final scores between groups.

### 3.8. Complications

Post-operative complications were reported in 8 out of 10 studies (80%), although the majority were minor and self-limiting. Across all studies, the most frequently reported outcome was the absence of complications, explicitly mentioned in four cases, confirming the overall safety of the procedure. Among the complications described, the most common included superficial wound dehiscence, scar retraction, range-of-motion limitation, transient sural nerve paresthesia, and one peri-implant fracture at the medial anchor insertion site (Table 3). These adverse events were generally managed conservatively and did not result in surgical revision. No deep infections, thromboembolic events, or permanent neurological deficits were reported in the included studies. Overall, despite the technical complexity of combined arthroscopic procedures, the complication rate remained low, and patient satisfaction—when reported—was high. Most patients were able to return to daily life and sporting activity without functional limitations.

## 4. Discussion

This systematic review investigated the current evidence on the arthroscopic treatment of medial and rotational ankle instability. The collective data demonstrate that arthroscopic repair is associated with excellent functional outcomes, a low complication rate, and a timely return to sport and daily activities. Across the included studies, the mean postoperative AOFAS score was 95.3, reflecting near-complete functional recovery in most patients. Notably, return to sport was commonly achieved between 3 to 5 months postoperatively, with one comparative study demonstrating a significantly faster return to play in patients undergoing arthroscopic deltoid ligament repair compared to non-repair groups (4.6 ± 1.6 vs. 6.0 ± 2.5 months, *p* = 0.03) [37].

Arthroscopic techniques offer multiple advantages over traditional open repair. First, minimally invasive access reduces soft tissue dissection, which is associated with less postoperative pain, faster rehabilitation, and superior cosmetic outcomes. The small portal incisions and avoidance of extensive dissection result in lower rates of wound complications, a point highlighted by our findings: no deep infections or reoperations were reported among the 336 patients included, and only minor complications—such as superficial wound issues or transient paresthesia—were observed. These rates are lower than those typically reported in open repair series, where complication rates range from 10% to 15%, including wound dehiscence and infections. Similar findings were reported by Krogsgaard et al., who noted that open deltoid repair, although commonly practiced, led to suboptimal long-term outcomes and more wound-related issues [38]. Kim et al. further supported the equivalency of arthroscopic and open repair outcomes, concluding that arthroscopic techniques may offer similar functional results with reduced soft tissue disruption [39].

Second, arthroscopy enables direct visualization and treatment of intra-articular pathologies, which are frequently present in patients with rotational instability. In fact, 90% of the studies in this review reported the need for at least one additional procedure—such as osteophyte resection, microfracture, or synovectomy—performed during the same session. These combined procedures are often more comprehensively addressed via arthroscopy than open surgery, which may require separate exposures or staged interventions. Vega et al. described the benefits of combining arthroscopic medial and lateral ligament repair in the same procedure, resulting in excellent outcomes with a significant increase in AOFAS scores (from 70 to 100) and no recurrence of instability at follow-up [26].

Biomechanical studies have further confirmed that arthroscopic repair techniques effectively restore ankle stability, particularly in valgus and rotational planes [32]. Moreover, the use of knotless suture anchors has simplified intra-articular ligament repair while minimizing the risk of hardware irritation. In support of this, Tansey et al. described an all-inside, knotless arthroscopic technique that offers improved biomechanical stability with minimal invasiveness, providing promising clinical outcomes in early studies [40]. Aicale et al. have emphasized that these techniques are particularly effective for treating rotational ankle instability, which involves subtle deltoid complex injuries often undetectable with standard imaging [41].

Although the steep learning curve has been acknowledged, this aspect deserves further discussion. Arthroscopic deltoid repair requires advanced skills and may not be readily applicable in low-volume or less experienced centers. Inadequate portal placement, limited visualization, or misinterpretation of ligament damage could increase the risk of suboptimal repair or iatrogenic injury. As such, the procedure may not be suitable for all surgeons or settings. Surgical decision-making should take into account institutional expertise, and the adoption of dedicated training programs or cadaveric simulation could help mitigate risks and expand the use of this technique safely.

The importance of surgeon experience and individualized surgical planning has been emphasized by Loozen et al., particularly in complex or recurrent instability [42].

Compared to historical open techniques—which are associated with longer recovery times, higher postoperative pain, and increased wound complications—arthroscopy represents a compelling alternative, particularly in young, athletic patients seeking early return to function [43]. While open repairs have reported mean AOFAS scores ranging from 78 to 92 in similar cohorts, the present review shows a consistently higher range (86 to 100), suggesting a potential clinical advantage of arthroscopy in selected patients.

Nevertheless, current evidence is limited by the absence of high-quality, prospective randomized trials comparing open and arthroscopic medial ligament repair. While retrospective series and technical reports show promising results, future studies should aim to clarify long-term outcomes, patient selection criteria, and cost-effectiveness. Moreover, as highlighted by Schrempf et al., the lack of standardized diagnostic criteria and imaging protocols for deltoid ligament injuries continues to limit early diagnosis and consistent treatment across centers [44].

### Study Limitations

This review has several limitations that should be acknowledged. First, most of the included studies were retrospective case series, with only a few prospective investigations and no randomized controlled trials. As such, the overall level of evidence is low, and the results must be interpreted with caution due to potential selection and reporting bias. Second, there was heterogeneity in the surgical techniques and rehabilitation protocols employed across studies, which limits the ability to perform direct comparisons or meta-analytic synthesis of outcomes.

Third, many studies included patients with combined medial and lateral instability and often performed additional procedures such as osteochondral debridement or syndesmotic fixation. This makes it difficult to isolate the clinical effect of the medial ligament repair itself. Additionally, preoperative diagnostic criteria for medial ankle instability were not standardized and often relied on intraoperative confirmation, introducing a degree of subjectivity in patient selection.

Follow-up duration varied widely among the studies, with some reporting outcomes at only 12 months. This may not be sufficient to evaluate the long-term durability of arthroscopic medial repairs or the risk of recurrent instability. Finally, although all studies reported functional outcomes using the AOFAS score, validated patient-reported outcome measures (PROMs), such as the Foot and Ankle Ability Measure (FAAM), the EQ-5D, or patient satisfaction scales, were largely absent. This omission limits the ability to capture the patient’s subjective perception of recovery, functional capacity, and overall quality of life. Future research should prioritize the use of standardized PROMs to ensure more comprehensive, patient-centered evaluation of treatment outcomes.

Future studies should aim to include larger, adequately powered sample sizes and adopt prospective, comparative study designs directly comparing arthroscopic and open techniques. The use of standardized diagnostic criteria, clearly defined inclusion parameters, and uniform surgical and rehabilitation protocols would enhance comparability across studies. Furthermore, follow-up durations should extend beyond 12 months to evaluate long-term outcomes, including recurrence of instability and functional durability. Lastly, validated PROMs such as the FAAM, EQ-5D, and satisfaction scores should be incorporated to ensure a more comprehensive and patient-centered assessment of treatment effectiveness.

## 5. Conclusions

Arthroscopic repair of medial and rotational ankle instability represents a safe, effective, and minimally invasive alternative to traditional open techniques. This systematic review demonstrates that arthroscopic management leads to excellent functional outcomes, low complication rates, and rapid return to sport, particularly in active and athletic populations. The ability to simultaneously diagnose and treat associated intra-articular lesions further supports the value of this approach.

Although promising, current evidence is limited by the predominance of non-comparative studies and methodological heterogeneity. High-quality prospective trials with standardized outcome measures are needed to better define the role of arthroscopy compared to open reconstruction, especially in complex or chronic instability cases. Until such evidence is available, arthroscopic repair should be considered a valuable option in selected patients, provided it is performed by experienced surgeons with adequate arthroscopic expertise.

## Figures and Tables

**Table 1:** Inclusion and exclusion criteria.

Study Aspect	Inclusion Criteria	Exclusion Criteria
Types of studies	1. Therapeutic clinical studies evaluating arthroscopic lateral ankle ligament repair 2. Studies with retrospective or prospective design 3. Randomized and quasi-randomized controlled trials 4. Studies reporting preoperative and postoperative outcome scores 5. Minimum 12-month follow-up for all included patients 6. Published in peer-reviewed journals 7. Written in English 8. Full-text available	1. Review articles 2. Case reports 3. Technique articles 4. Cadaveric studies 5. Animal studies 6. In vivo basic science studies
Types of participants	1. Adult patients (≥18 years old) diagnosed with medial or rotational ankle instability 2. Patients undergoing arthroscopic ligament repair as primary or combined procedure	1. Pediatric populations (<18 years old) 2. Patients treated non-operatively 3. Cases involving experimental or non-standard interventions
Types of interventions	1. Arthroscopic surgical procedures for the treatment of medial ankle instability (including deltoid ligament repair or combined repairs)	1. Open or hybrid procedures without an arthroscopic component 2. Non-surgical interventions 3. Experimental techniques not supported by outcome data

**Table 2:** Demographic data. ns: not specified.

Authors	Type of Study	Number of Patients	Number of Ankles	Male	Female	Mean Age (Year)	Follow-Up (Month)
Mansur et al. [25]	Retrospective	29	30	14	15	38	14.8
Vega et al. [26]	Retrospective	7	7	ns	ns	23	34
Vega et al. [23]	Retrospective	13	13	12	1	32	35
Buchhorn et al. [19]	Prospective	81	81	30	51	31.9	12
Seiça et al. [27]	Retrospective	39	39	23	16	40.1	21.3
Li et al. [28]	Retrospective	24	24	20	4	33	28
Lewis et al. [29]	Prospective	12	12	6	6	33.9	21
Hintermann et al. [30]	Prospective	51	52	27	25	36.4	52
Vega et al. [31]	Prospective	8	8	7	1	31	22
Guelfi et al. [32]	Prospective	25	25	16	9	27	24
Total		289	291	155	128	32.6	26.4

**Table 3:** Management of medial or rotational ankle instability; AOFAS: American Orthopaedic Foot and Ankle Society, ATFL: anterior talofibular ligament, ns: not specified.

Authors	Conservative Approach (Yes/No)	Duration of Conservative Approach (Month)	Surgical Approach	Additional Surgery	AOFAS SCORE	Complications
Mansur et al. [25]	Yes	6	Arthroscopic all-inside medial and lateral ligament repair with a knotless suture anchor technique	Microfracture for osteochondral lesion of the talus peroneal tendoscopy Lateral internal bracing syndesmosis fixation	91.9	1 peri-implant fracture 1 transient paresthesia 1 superficial infection 1 scar retraction
Vega et al. [26]	Yes	ns	Arthroscopic all-inside medial and lateral ligament repair with a knotless suture anchor technique	Chondral talar lesion debridement [1] Osteophyte resection [5] Hindfoot endoscopy [3]	100	None
Vega et al. [23]	Yes	ns	Arthroscopic all-inside medial and lateral ligament repair with a knotless suture anchor technique	Osteochondral talar lesion debridement and microfracture [2] Osteophyte resection [1] Posterior endoscopy [2]	100	None
Buchhorn et al. [19]	Yes	ns	Arthroscopic all-inside medial and lateral ligament repair with a knotless suture anchor technique	ns	94.1	4 delayed wound healing 1 hyperesthesia
Seiça et al. [27]	Yes	ns	10: Arthroscopic all-inside medial and lateral ligament repair with a knotless suture anchor technique; 10: Retensioning procedures	ns	86.9	1 superficial wound dehiscence 4 ROM limitation 1 skin retraction 1 dysesthesia
Li et al. [28]	Yes	6	Arthroscopic all-inside medial and lateral ligament repair with a knotless suture anchor technique	ns	98	None
Lewis et al. [29]	Yes	ns	Arthroscopic all-inside medial and lateral ligament repair with a knotless suture anchor technique	ns	ns	None
Hintermann et al. [30]	Yes	7.1	Arthroscopic all-inside medial and lateral ligament repair with a knotless suture anchor technique, augmentation with plantaris tendon graft if needed	Calcaneal lengthening osteotomy	91.6	None
Vega et al. [31]	Yes	3	Arthroscopic anterior deltoid plication with a bony anchor	Synovectomy and osteophyte resection [2]	100	None
Guelfi et al. [32]	Yes	6	Arthroscopic all-inside lateral and medial ligament repair with a knotless suture anchor technique	ATFL repaired with a knotless anchor	ns	None

## Data Availability

All of the data we analyzed and the tables we compiled are available for any clarification.

## Abbreviations

ATFL	Anterior talofibular ligament
CFL	Calcaneofibular ligament
CAI	Chronic ankle instability
RAI	Rotational ankle instability
MCL	Medial collateral ligament
MRI	Magnetic resonance imaging
AOFAS	American Orthopaedic Foot and Ankle Society
PROMs	Patient-reported outcome measures
EQ-5D	EuroQol-5 Dimensions
FAAM	Foot and Ankle Ability Measure
MINORS	Methodological Index for Non-Randomized Studies
ROM	Range of motion
IRB	Institutional Review Board
PRISMA	Preferred Reporting Items for Systematic Reviews and Meta-Analyses
